# Intestinal decolonization of *Enterobacteriaceae* producing extended-spectrum β-lactamases (ESBL): a retrospective observational study in patients at risk for infection and a brief review of the literature

**DOI:** 10.1186/s12879-015-1225-0

**Published:** 2015-10-28

**Authors:** Siegbert Rieg, M. Fabian Küpper, Katja de With, Annerose Serr, Jürgen A. Bohnert, Winfried V. Kern

**Affiliations:** Division of Infectious Diseases, Department of Medicine, Albert-Ludwigs-University Hospital & Medical Center, Hugstetter Straße 55, D-79106 Freiburg, Germany; Center for Chronic Immunodeficiency, Albert-Ludwigs-University Hospital & Medical Center, Freiburg, Germany; Department of Microbiology, Albert-Ludwigs-University Hospital & Medical Center, Freiburg, Germany; Present address: Clinical Infectious Diseases and Antibiotic Stewardship Unit, Carl Carus University Hospital, Dresden, Germany; Present address: Institute of Medical Microbiology, University Hospital, Jena, Germany

**Keywords:** ESBL, Eradication, Decontamination, Colistin, Rifaximin, Paromomycin

## Abstract

**Background:**

Multidrug-resistant *Escherichia coli* and other enteric bacteria producing extended-spectrum β-lactamases (ESBL) have emerged as an important cause of invasive infection. Targeting the primary (intestinal) niche by decolonization may be a valuable approach to decrease the risk of relapsing infections and to reduce transmission of ESBL-producing enteric pathogens.

**Methods:**

In a retrospective observational study we evaluated the efficacy of intestinal decolonization treatment using orally administered colistin or other non-absorbable agents given for 2 to 4 weeks in adult patients with previous relapsing infection and persistent intestinal colonization with ESBL-positive *Enterobacteriaceae* (ESBL-E). Eradication success was defined as negative rectal swab or stool culture at the end of treatment and at follow up-2 weeks after treatment discontinuation.

**Results:**

First-line decolonization treatment led to eradication of ESBL-E in 19/45 patients (42 %, 7/18 low-dose [4 × 1 million units] colistin, 3/12 high-dose [4 × 2 million units] colistin, 9/15 rifaximin [2 × 400 mg]), and secondary/salvage treatment was successful in 8/13 patients (62 %, 20 treatment episodes). Late follow-up showed that 7/13 patients (54 %) with successful initial or salvage decolonization became recolonized within 3 months after post-treatment assessment while all eight of the patients failing initial or salvage decolonization treatment with late follow-up remained colonized. A narrative review of the literature confirms the limited efficacy of non-absorbable antibiotics including conventional selective digestive tract decolonization (SDD)-like combination regimens for eradicating multidrug-resistant enteric bacteria from the intestinal tract.

**Conclusions:**

At present, there is no clear evidence of a significant decolonization efficacy using single-drug treatment with oral non-absorbable antibiotics. More effective regimens are needed and a better definition of at risk patients is required for planning meaningful randomized controlled studies in this field.

## Background

Extended-spectrum β-lactamase (ESBL)-producing gram-negative *Enterobacteriaceae* (ESBL-E) are emerging pathogens [1]. The incidence of infection due to such microorganisms has been increasing substantially over the last years [2]. Treatment options in severe infection are limited, and many infectious diseases practitioners and medical microbiologists are concerned about the resulting overuse of carbapenems exerting a selection pressure leading to the emergence of carbapenemase-producing microorganisms [3, 4].

Patients with infection due to ESBL-E and other enteric bacteria resistant to third-generation cephalosporins are usually colonized by these bacteria in the intestinal tract. Spontaneous loss of ESBL-positive colonizers does occur, but is substantially delayed in case of repeated hospitalizations and antibiotic therapies [5]. Regimens for decolonization of ESBL-E in the intestinal tract have been proposed. The experience with such regimes, however, is limited, and the evidence for their efficacy comes from few studies most of which are observational. It is unknown which regimen is best suited and best tolerated, what is the optimum dose and the minimum treatment duration to ensure sustained eradication and not only suppression of the ESBL-positive organisms in the gut. On the other hand there is vast experience with and substantial clinical trial data for so-called selective digestive tract decolonization (SDD) regimens in intensive care patients and in neutropenic cancer patients in whom preventive treatment with nonabsorbable drugs like colistin plus tobramycin can decrease the risk of infection due to gram-negative enteric bacteria [6]. In addition, other nonabsorbable drugs like rifaximin have been used successfully for suppression of pathogenic or potentially pathogenic enteric bacteria and thereby may yield clinically beneficial effects in travellers’ diarrhea, hepatic encephalopathy and Crohn´s disease [7].

We started to offer individualized management to patients with a history of repeated infection due to ESBL-E. Patients with documented relapsing infection referred to our specialized outpatient clinic were screened for intestinal tract colonization with ESBL-E and if positive and remaining at risk for infection were offered decolonization treatment with oral colistin or alternative regimens. Initially this programme was offered as an infection control quality improvement programme to renal transplant patients but subsequently was open to other patients with relapsing infection due to cephalosporin-resistant enteric bacteria. In the present paper we summarize our experience with intestinal ESBL-E decolonization regimens in 45 patients given either colistin or rifaximin as first-line regimen and a variety of salvage treatments in case of failure. We discuss the results in the light of other similar data from the literature that show limited efficacy of current regimens in eradicating multidrug-resistant enteric bacteria from the intestinal tract.

## Methods

### Setting, study design and patient eligibility

The University Medical Center Freiburg is a tertiary care center in Southwestern Germany with 1.500 beds (65.000 inpatient cases per year) and 550.000 outpatient visits per year. The study was conducted in accordance with the standards set by the Declaration of Helsinki and the research guidelines of the University of Freiburg. We did a retrospective analysis of patients transferred between November 2008 and September 2012 to our infectious diseases outpatient department because of relapsing infection due to ESBL-E for advice on options for prevention of further infections. The patients with a history of at least two invasive ESBL-E infections [urinary tract infection, bloodstream infection, wound infection, respiratory tract infection or other sites] within the last year were screened for persistent colonization by ESBL-E by culture of rectal swabs and/or fecal samples, urine and other specimens depending on information on previous sites of colonization or infection. Patients with continued intestinal colonization by ESBL-positive bacteria resistant to fluoroquinolones and trimethoprim-sulfamethoxazole or who were intolerant to these drugs were offered individualized treatment with nonabsorbable antibiotics as a decolonization trial. All patients gave verbal or written informed consent before treatment. The local ethics committee (Ethical Committee of the University of Freiburg Medical Center) approved this retrospective analysis of patient outcomes.

### Decolonization treatment

Regimens initially consisted of oral colistin 4 × 1 million units daily for 4 weeks. After the impression of poor results with this regimen, we decided to increase the colistin dose to 4 × 2 million units daily (for 4 weeks) (“high-dose”) or to prescribe an alternative initial regimen of rifaximin (2 × 400 mg daily for 2 to 3 weeks) on the discretion of the infectious disease physician in charge.

Patients with positive urine cultures were given additional treatment with oral fosfomycin (3 g single-dose), nitrofurantoin (2 × 100 mg daily for 5 days), or with cefpodoxime (2 × 100 mg daily) plus amoxicillin-clavulanic acid (2 × 875/125 mg daily) or a carbapenem (renal-function adjusted dose, for 3 to 7 days).

### Patient follow-up and definition of success

The patients were asked to present for follow-up 12–16 days (colistin) or 5–10 days (rifaximin) after treatment initiation, at the end of treatment and 2 weeks after treatment discontinuation. Follow-up investigations included a physical work-up, assessment of tolerance and possible adverse events, a rectal swab for ESBL-E and additional clinical specimens for culture if other sites had been positive before. For the purpose of this analysis decolonization success was defined as negative cultures from rectal swabs or stool at the end of therapy and 2 weeks after therapy discontinuation. Patients without negative cultures during decolonization treatment were considered “primary failures”. For patients failing, treatment options were a repeated course of the initially prescribed regimen or a change to oral paromomycin (3 × 500 mg daily), rifaximin (dosage and duration as above), or watch and wait. During further (late) follow-up (>3 months after the last 2-week post-treatment assessment) cases were analysed for new cultures positive for ESBL-E by assessing microbiology reports in the hospital clinical information system.

### Microbiology

Clinical specimens and rectal swabs were processed at the local microbiology department in accordance with “Microbiology Procedures Quality Standards (MiQ)” issued by the German Society for Hygiene and Microbiology. ChromID ESBL (BioMérieux, Lyon, France) was used as commercially available chromogenic screening medium to detect ESBL-producing *Enterobacteriaceae*. The microorganisms were identified by standard microbiological techniques using Vitek 2 (BioMérieux, Germany) or MALDI-TOF mass spectrometer (Bruker Daltonic, Germany). Susceptibility testing including confirmatory tests for ESBL identification were carried out with Vitek 2. Additional confirmatory tests for ESBL production were performed using double-disc synergy test using ceftazidime, cefotaxime and cefpodoxime with and without clavulanic acid on Muller Hinton agar supplemented with cloxacillin where appropriate. Minimal inhibitory concentrations (MICs) were determined by gradient diffusion (Etest, BioMérieux, Germany) according to the manufacturer’s instructions. In selected microorganisms additional MICs of a panel of different drugs were determined by microdilution tests according to standard protocols and EUCAST interpretive guidelines.

### Statistical analysis

Groups of interest were compared by binomial tests or Fisher’s exact test as appropriate. A (two-sided) *p*-value of <0.05 was considered significant. Analyses were performed using SPSS Statistics version 20 (SPSS Inc., Chicago, IL, USA).

### Literature search

A literature search was performed in PubMed (through January 2015) by using the term (ESBL[All Fields] AND decolonization[All Fields]) OR (SDD[All Fields] AND resistance[All Fields]) and by additional handsearching of original articles.

## Results

### Patient characteristics and microbiology

A total of 45 patients (out of 55) completed all follow-up visits and were evaluable for the present analysis. The median age was 57 years (range, 19 to 86), 20 patients were male, and 25 patients were female. Many patients were renal transplant recipients but there was a variety of other underlying diseases (Table 1). Relapsing infections in the previous year were primarily urinary tract infections. The most frequent microorganism was *E. coli*, followed by *K. pneumoniae* (Table 1). Most isolates were resistant to fluoroquinolones, trimethoprim-sulfamethoxazole and tetracycline. All were susceptible to carbapenems, and no or very few *E. coli* isolates were resistant to fosfomycin and nitrofurantoin, respectively, while resistance to gentamicin was observed in 21 *E.coli* isolates (55 %, Table 1) and in three of the nine *K. pneumoniae* isolates (33 %).

**Table 1:** Clinical and microbiological characteristics of 45 patients according to first-line decolonization results

Parameter	Total	Decolonization success	Decolonization failure
	(*n* = 45)	(*n* = 19)	(*n* = 26)
Median age (range), yrs	57 (19–86)	56 (19–84)	60 (20–86)
Male, n (%)	20 (44 %)	9 (47 %)	11 (42 %)
Underlying diseases, n (%)			
▪ Renal transplant	12 (27 %)	5 (26 %)	7 (27 %)
▪ Other solid organ transplant	1 (2 %)	1 (5 %)	-
▪ Autoimmune/collagen vascular disease	4 (9 %)	1 (5 %)	3 (12 %)
▪ Obstructive uropathy	16 (36 %)	5 (26 %)	11 (42 %)
▪ Nephrolithiasis	1 (2 %)	1 (5 %)	-
▪ Lymphoma or cancer	6 (13 %)	4 (21 %)	2 (8 %)
▪ CVID	2 (4 %)	1 (5 %)	1 (4 %)
▪ Other	11 (24 %)	4 (21 %)	7 (27 %)
Previous infections, n (%)			
▪ Urinary tract infection	38 (84 %)	16 (84 %)	22 (85 %)
▪ Bloodstream	9 (20 %)	4 (21 %)	5 (19 %)
▪ Wound infection	5 (11 %)	3 (16 %)	2 (8 %)
▪ Respiratory tract infection	2 (4 %)	1 (5 %)	1 (4 %)
▪ other sites	2 (4 %)	1 (5 %)	1 (4 %)
Microorganisms, n (%)			
▪ *E.coli*	29 (64 %)	11 (58 %)	18 (69 %)
▪ *E.coli* plus other	9 (20 %)	5 (26 %)	4 (15 %)
▪ *Klebsiella pneumoniae*	6 (13 %)	2 (11 %)	4 (15 %)
▪ *Enterobacter aerogenes*	1 (2 %)	1 (5 %)	-
In vitro resistance (*E.coli*) (*n* = 38), n (%)			
▪ Ciprofloxacin	33 (87 %)	13 (81 %)	20 (91 %)
▪ Trimethoprim-sulfamethoxazole	35 (92 %)	14 (88 %)	21 (95 %)
▪ Gentamicin	21 (55 %)	7 (50 %)	14 (64 %)
▪ Tetracycline	33 (87 %)	14 (88 %)	19 (86 %)
▪ Fosfomycin	-	-	-
▪ Nitrofurantoin	2 (5 %)	-	2 (9 %)
Decolonization regimen, n (%)			
▪ Colistin 4 × 1	18 (40 %)	7 (37 %)	11 (42 %)
▪ Colistin 4 × 2	12 (27 %)	3 (16 %)	9 (35 %)
▪ Rifaximin	15 (33 %)	9 (47 %)	6 (23 %)
Initial additional UTI treatment, n (%)	26 (58 %)	8 (42 %)	18 (69 %)
▪ Oral fosfomycin	9 (20 %)	2 (11 %)	7 (27 %)
▪ Oral nitrofurantoin	3 (7 %)	1 (5 %)	2 (8 %)
▪ Oral cefpodoxime + amoxi-clav	7 (16 %)	4 (21 %)	3 (12 %)
▪ Parenteral carbapenem	7 (16 %)	1 (5 %)	6 (23 %)

*Yrs* years, *n* numbers, *CVID* common variable immunodeficiency syndrome, *UTI* urinary tract infection, *colistin* daily dosage given in million units

Twenty of the 38 *E.coli* and three of the nine *K. pneumonia*e isolates were retested for MICs in a microdilution assay. These tests confirmed the results of the routine susceptibility tests and revealed high rates of resistance in *E.coli* to streptomycin (MIC >32 μg/mL, 17 isolates [85 %]) and to azithromycin (MIC >16 μg/mL, all isolates), but low rates of resistance to tigecycline (MIC >0.5 μg/mL, no isolate), to chloramphenicol (MIC >8 μg/mL, 3 isolates [15 %]), and to paromomycin (MIC >32 μg/mL, 3 isolates [15 %]). The geometric mean MIC of rifaximin was 64 μg/mL (range, 16–128 μg/mL), and the geometric mean MIC of colistin was 0.5 μg/mL (range, 0.125–1 μg/mL), respectively.

The three *K. pneumoniae* isolates tested for MICs were resistant to streptomycin, chloramphenicol, tigeycline, azithromycin and paromomycin, and, compared with *E.coli*, showed higher MICs of colistin (2–4 μg/mL) but similar MICs of rifaximin (64–128 μg/mL) (data not shown).

### Outcomes of initial treatment

Successful decolonization after initial treatment was observed in 19 patients (42 %, 95 % confidence interval 23–56 %). Treatment with colistin in either dosage yielded lower rates than rifaximin (low-dose colistin, 7/18 [39 %]; high-dose colistin, 3/12 [25 %]; rifaximin, 9/15 [60 %]), but the differences were not statistically significant. Patients with successful decolonization were slightly younger, had more often malignancies as underlying disease but less often obstructive uropathy and needed less often additional antibiotics for treatment of urinary tract infection or bladder colonization (Table 1). Moreover, the microorganisms showed slightly higher rates of resistance to some of the tested drugs in patients with treatment failures (Table 1), but all these differences were statistically not significant.

Among the 26 patients without successful decolonization, nine were considered primary failures (18 % of all, 95 % confidence interval 8–32 %) while the other 17 patients with failure had negative cultures during decolonization but became rapidly positive after treatment discontinuation. One patient (considered a treatment failure) had initial intestinal colonization with *E. coli* and *K. oxytoca* and became negative for *E.coli* but remained positive for *K. oxytoca* during and after treatment with colistin. The proportions of patients with primary failures were not different between the three treatment groups.

Among the organisms cultured from patients with failure we found one *E.coli* with a colistin MIC of 2 μg/mL, possibly indicating development of resistance during treatment since the original strain was fully susceptible to colistin but unavailable for retesting and typing. All other isolates from failures that were available for retesting (*n* = 11) did not show changes in the MIC of colistin or rifaximin.

Following treatment, 14 patients in the failure group (54 %, 95 % confidence interval 35–73 %) versus none in the success group had positive urine cultures with the initially colonizing strains, and this appeared to be independent of the type of the additional urinary tract infection (UTI) antibiotics prescribed.

### Salvage regimens and late follow-up

A total of 13 patients failing the initial regimen were subsequently given salvage intestinal decolonization regimens for a total of 20 treatment episodes. Eight of the patients (62 %, 95 % confidence interval 41–83 %) were eventually cleared of ESBL-E. After the first salvage regimen only 5/13 were successes. Four patients received a second salvage therapy (one success), and three patients received a third salvage regimen (two successes). Thus, when considering initial and salvage regimens, 27/45 patients (60 %, 95 % confidence interval 46–74 %) were successfully decolonized according to results of early follow-up swabs (2 weeks post-treatment).

As salvage regimens a variety of regimens were given. These included colistin (standard dose, four patients; high-dose, three patients), rifaximin (six patients), or paromomycin (seven patients). Success rates were 3/7 with colistin (43 %), 3/6 with rifaximin (50 %), and 2/7 with paromomycin (29 %).

According to microbiology records there were 21 cases with late follow-ups (>3 months after the last 2-week post-treatment assessment) that included rectal swab and stool cultures (and other clinical cultures in many cases). In this subgroup, three out of seven patients with initially successful intestinal decolonization became culture-positive during late follow-up (corresponding to three relapses during 9.4 person years of observation) while this figure was 3/6 for the patients with initial failures but successful salvage (three relapses during 7.6 person years) which corresponds to a rate of 7/13 for sustained decolonization (54 %). Of the eight patients with unsuccessful first salvage regimens all remained or became positive during late follow-up (covering 4.8 person years) although some had intermittently been negative (including one success after second salvage).

### Review of the literature

**Fig. 1:**
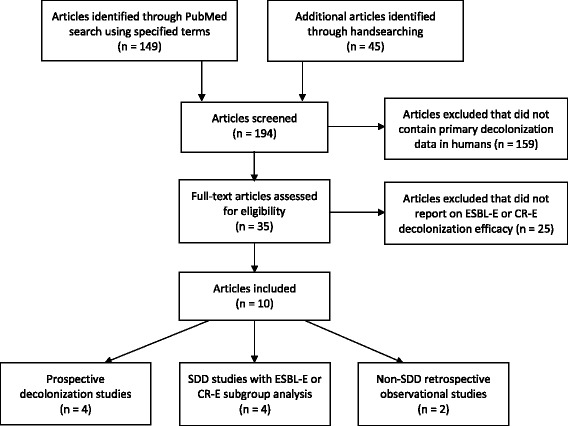
Flow diagram of literature search. ESBL-E extended-spectrum β-lactamase-producing Enterobacteriaceae, CR-E carbapenem-resistent Enterobacteriaceae, SDD selective digestive tract decolonization

The initial search using the indicated terms yielded 149 articles, handsearching identified 45 additional articles. Of the resulting 194 articles that were screened 35 full-text articles were assessed in detail for eligibility (Fig. 1). After exclusion of studies that did not allow extraction of data on ESBL-E or carbapenem-resistent *Enterobacteriaceae* (CR-E) decolonization efficacy, ten articles were included in the current review. Among these, four prospective studies were identified that primarily aimed to investigate decolonization of ESBL-E or CR-E. In a randomized, double-blind placebo-controlled trial Huttner et al. found a transient lower carriage rate of ESBL-E at day 1 after decolonization using a 10 day regimen of colistin and neomycin [8]. However no significant difference in the primary study endpoint (decolonization rate/negative rectal swab 4 weeks after the end of treatment) were observed with successful decolonization in 52 % of patients with active treatment versus 37 % in the placebo group. Saidel-Odes et al. described successful CR-E decolonization in the first week after a 7 day regimen of colistin plus gentamicin (61 % vs. 16 % in placebo arm) but, again, this significant effect did not last until the follow-up at 6 weeks (59 % vs. 33 % in placebo arm) [9]. Oren et al. observed a 44 % efficacy in eradicating CR-E using colistin and/or gentamicin. This was reported to be significantly different to the spontaneous eradication rate, yet the latter was with 7 % very low [10]. Buehlmann et al. investigated ESBL-E decolonization using a 4 day course of paromomycin in intestinal carriage and oral antibiotics in urinary tract colonization [11]. As >80 % of patients suffered from ESBL-E infection (with a non-reported intestinal colonization rate) and more than half of the subjects cleared ESBL-E carriage without the specified decolonization protocol (i.e. by systemic antimicrobial and/or surgical treatment), the high decolonization success rate of 76 % should be interpreted cautiously.

Four SDD studies were identified that reported on ESBL-E or CR-E decolonization efficacy within subgroup analyses: Lübbert et al. observed no significant differences with regard to decolonization rates between patients receiving 7 days of a colistin and gentamicin-containing SDD-regimen (59 % SDD group vs. 33 % in control group) in follow up-cultures after 6 weeks [12]. Using colistin plus tobramycin until intensive care unit (ICU) discharge, Oostdijk et al. found higher decolonization rates for 3rd generation cephalosporin-resistant *Enterobacteriaceae* (73 %) and for aminoglycoside-resistant *Enterobacteriaceae* (62 %). However, as patients were not followed up, this reflects end of treatment efficacy, and no sustained effects were reported [13]. The same holds true for the studies of Abecassis et al. and of Troché et al. with ESBL-E clearance under SDD regimens of 54 and 46 % respectively [14, 15].

Finally, two retrospective observational non-SDD studies were identified. Nitschke et al. describe a high efficacy of azithromycin in shortening the duration of intestinal shedding of ESBL-positive, shiga-toxin producing, enteroaggregative *E. coli* (STEC) O104:H4 [16]. These data are derived from the specific setting of an STEC outbreak with associated hemolytic uremic syndrome in Germany in 2011. With regard to the causative pathogen and the patients included comparability to the results of the other studies seems limited. The second study reported predominantly transient suppression of ESBL-E after a 5 day course of norfloxacin [17].

A summary of the relevant studies containing information on included patients, targeted pathogen, decolonization regimen and efficacy as well as resistance development is available in Table 2.

**Table 2:** Identified studies in the context of ESBL-E or CR-E decolonization

Author, year; study type	Patients included	Pathogen	Decolonization regimen^a^ [duration]	Decolonization efficacy [definition, methods to detect]	Follow-up period	Resistance development	Remarks
Prospective ‘decolonization’ studies							
Huttner et al., 2013; randomized, double-blind, placebo-controlled study [8]	Hospitalized carriers (*n* = 54)	ESBL-E (*E. coli* ~80 %, *K. pneumoniae* ~20 %)	Colistin 1,26 MU, neomycin 250 mg [10d] (plus nitrofurantoin 3 × 100 mg/d [5d] in urinary tract colonization) (*n* = 27) vs. placebo (*n* = 27)	52 % vs. 37 %; no significant difference [≥1 neg. rectal swab culture]	28 ± 7 days	No significant change in colistin or neomycin MICs	Extraintestinal colonization 50 % at baseline; systemic antibiotic treatment in 4 % of patients (vs. 19 % in placebo group)
Oren et al., 2013; prospective, controlled study [10]	Hospitalized carriers (*n* = 152), ~40 % of which with clinical infection	CR-E (*K. pneumoniae* ~90 %)	Colistin 2,5 MU (*n* = 16) or gentamicin 80 mg (*n* = 26) or combination (*n* = 8) [until decolonization, max. 60 d] vs. spontaneous eradication (*n* = 102)	44 % (42 %, 50 %, 37.5 %) vs. 7 %; significant difference [3 neg. consecutive rectal swabs cultures, neg. PCR testing of third swab]	Median f/u 33 days vs. 140 days	Gentamicin resistance 23 %; colistin resistance 6 %, combination 0 %	Systemic antibiotic treatment in ~40 % of patients in both groups
Saidel-Odes et al., 2012; randomized, double-blind, placebo-controlled study [9]	Hospitalized carriers (*n* = 40)	CR-E (*K. pneumoniae*)	Colistin 1 MU, gentamicin 80 mg, plus SOD [7d] (*n* = 20) vs. placebo (*n* = 20)	59 % vs. 33 %; no significant difference [neg. rectal swab culture]	42 days	None, gentamicin MIC remained ≤2 mg/ml and colistin MIC ≤0.094 mg/ml	Efficacy 61 % vs. 16 % at week 2; no impact on extraintestinal colonization (groin cultures 60 % positive)
Buehlmann et al., 2011; prospective, controlled study [11]	Infected patients (*n* = 83) or hospitalized carriers (*n* = 17)	ESBL-E (*E. coli* 71 %, *K. pneumoniae* 25 %)	Paromomycin 1 g (intestinal colonisation) [4d], diverse oral antibiotics (urinary tract colonization) [5d], chlorhexidine mouth rinse [5d] (*n* = 39) vs. spontaneous eradication (*n* = 61)	63 % (ITT analysis)/83 % (on treatment analysis) vs. 55 % [≥1 neg. throat and rectal swab and neg. urine culture]	Median f/u 24 months	n.d.	55 % of patients eliminated ESBL-E without decolonization regimen by systemic antibiotic treatment or surgery
SDD studies with ESBL-E or CR-E decolonization efficacy subgroup analysis or retrospective observational studies							
Lübbert et al., 2013; retrospective, observational SDD study [12]	Hospitalized carriers (*n* = 52) or infected patients (*n* = 38)	CR-E (*K. pneumoniae*)	Colistin 1 MU, gentamicin 80 mg plus SOD [7d] (*n* = 14) vs. non SDD-control (*n* = 76)	43 % vs. 30 %; no significant difference [≥3 consecutive negative rectal swab PCRs separated by ≥48 h from one another]	Median f/u 48 days vs. 53 days	2/6 previous sensitive isolates acquired colistin resistance; 5/11 acquired gentamicin resistance	Systemic antibiotic treatment in 43 % of SDD group vs. 29 % non-SDD group
Oostdijk et al., 2012; post hoc subgroup analysis of prospective, randomized SDD study [13]	Hospitalized (ICU) carriers (*n* = 507)	3CR-E or AGR-E^b^	Colistin 2,5 MU, tobramycin 80 mg, amphotericin B 500 mg plus SOD [until discharge]; no control group	73 % in 3CR-E (vs. 80 % in cephalosporin-sensitive isolates), 62 % in AGR-E(vs. 81 % in aminoglycoside-sensitive isolates) [2 consecutive rectal swab cultures]	Until ICU discharge	No significant resistance development in patients with decolonization failure	Decolonization after median duration of 5 days in 3CR-E, 5.5 days in AGR-E (vs. 4 days in respective sensitive isolates)
Abecasis et al., 2011; post hoc subgroup analysis of prospective SDD study [14]	Hospitalized (pediatric ICU) carriers (*n* = 28) or infected patients (*n* = 11)	ESBL-E	Colistin, tobramycin, parenteral cefotaxime [until ICU discharge, dose and duration not specified]; no control group	Overall 54 % (21/39), with follow-up 21/27 (78 %) [negative rectal swab culture]	Until ICU discharge	No tobramycin resistence development	In 9/23 patients with tobramycin-resistent isolates decolonization failed (vs. 0/16 with tobramycin-sensitive isolates)
Troché et al., 2005; post hoc subgroup analysis of prospective SDD study [15]	Hospitalized (ICU) carriers (*n* = 27) or infected patients (*n* = 10)	ESBL-E	Colistin 1.5 MU plus neomycin 500 mg or plus erythromycin 500 mg [until two negative rectal swabs or ICU discharge]; no control group	46 % [2 consecutive negative rectal swab cultures]	Until ICU discharge	n.d.	Systemic antibiotic treatment in ten infected patients
Nitschke et al., 2012; retrospective observational cohort study^c^ [16]	Patients with intestinal carriage of STEC with or without HUS (*n* = 65)	STEC O104:H4	Azithromycin [cumulative 3 g in 14 days] (*n* = 22) vs. spontaneous eradication (*n* = 43)	95 % vs. 19 %; significant difference [≥2 neg. stool cultures over a period of at least 6 days]	Mean f/u 41 days vs. 45 days	n.d.	Subsequently, 15 long term STEC carriers were treated with 3 days azithromycin with 100 % decolonization efficacy
Paterson et al., 2001; retrospective observational cohort study^c^ [17]	Hospitalized carriers (*n* = 7) or infected patients (*n* = 2)	ESBL-E (*E. coli* 67 %, *K. pneumoniae* 33 %)	Norfloxacin 2 × 400 mg/d [5d]; no control group	44 % [≥1 neg. stool culture]	28 days	n.d.	Transient ESBL-E suppression at day 2–3 after completion of norfloxacin in 9/9 patients

*ESBL-E* extended-spectrum β-lactamase-producing Enterobacteriaceae, *CR-E* carbapenem-resistent Enterobacteriaceae, *3CR-E* 3rd generation cephalosporin-resistant Enterobacteriaceae, *AGR-E* aminoglycoside-resistant Enterobacteriaceae, *STEC O104:H4* shiga toxin–producing enteroaggregative *Escherichia coli*, *HUS* hemolytic uremic syndrome, *ICU* intensive care unit, *n* numbers, *n.d*. not determined, *neg*. negative, *SDD* selective digestive tract decolonization, *SOD* selective oral decontamination, *MU* million units, *f/u* follow-up, *MIC* minimal inhibitory concentration

^a^Decolonization regimens were applied four times daily via oral route or nasogastric tube unless otherwise stated as dose per day (/d). ^b^Resistance against ceftazidime, cefotaxime and ceftriaxone were considered as proxy for ESBL production. ^c^Not considered a SDD study

## Discussion

Apart from representing a major risk factor for invasive infection [18], carriage of ESBL-E may contribute to the spread of these multidrug-resistant and difficult-to-treat bacteria due to person-to-person-transmission and horizontal transfer of resistance genes to coresiding intestinal bacteria [19].

Accumulating evidence suggests prolonged intestinal carriage of ESBL-E. Follow-up of healthy travelers that were colonized with ESBL-E after their return revealed that only about half (54 %) of travelers cleared colonization within 2 months and at least 18 % of travelers remained colonized at reevaluation after 6 months (8 % of patients were lost to follow-up) [20]. Several studies reported persistent colonization of ESBL-E or CR-E after hospital discharge or at readmission in 60–90 % of patients after three months and 25–40 % after 12 months with repeated hospitalizations, transfer from other health care facilities, antibiotic (re)exposure and history of invasive disease (in contrast to detection in surveillance cultures only) extending the duration of colonization [5, 21–23].

The experience with intestinal decolonization in patients carrying ESBL-positive bacteria is limited. We and others argued that previous studies with SDD in intensive care and cancer patients can be regarded as proof-of-principle for the efficacy to prevent infection by topical decolonization or at least suppression of potentially pathogenic enteric bacteria. In fact, as shown in our review several investigators have published their experience with classical SDD or similar regimens in eradicating ESBL-E or CR-E from the intestinal tract. Decolonization efficacy in most of those studies was in the range of 40–50 % - whether this is significantly higher than the spontaneous eradication rate and a ‘watch and wait’ strategy cannot be answered by available data. Interpretation of the published series is particularly hampered by diverse (often very short) follow-up periods, different definitions of decolonization success and detection methods (additional methodological heterogeneities are summarized in Table 3).

**Table 3:** Methodological heterogeneities in previous studies and open questions for future decolonization studies

Methodological heterogeneities in previous studies
▪ Different ESBL-E sampling (perianal vs. rectal swab vs. fecal sample) and detection (culture vs. PCR-based technology vs. combined)
▪ Diverse definitions of decolonization success (number of negative samples, duration of follow-up period)
▪ In- or exclusion of patients with concomitant ESBL-E infection
▪ Intestinal decolonization with or without systemic antibiotic treatment
▪ Availability of pre-decolonization antibiotic susceptibility tests and variable impact on decolonization regimen
Open questions that need to be addressed in future studies
▪ Are there effective decolonization strategies leading to sustained clearance of ESBL-E?
▪ What is the optimal regimen (combination regimen?), dose and duration?
▪ Will we observe resistance development (and risk to lose important last resort antibiotics e.g. colistin)?
▪ Which patients may have the greatest benefit of decolonization?
▪ What is the impact of extraintestinal colonization (perianal region, groin)? Should decolonization strategies address this?
▪ Does relapse represent intestinal ‘outgrowth’ of suppressed ESBL-E or re-colonization from extraintestinal sites or other patients, food sources or the environment?
▪ Do pathogens differ with respect to the decolonization success rate (e.g. *Klebsiella* spp. vs. *Escherichia coli* vs. *Enterobacter* spp.)?
▪ How robust is the intestinal microbiome under antibiotic treatment? What is its impact on ESBL-E colonization resistance?

Of the four prospective studies two were randomized, double-blind, placebo-controlled trials. Both failed to demonstrate a significant difference in decolonization efficacy after 4–6 weeks using colistin in combination with an aminoglycoside when compared to placebo [8, 9]. Given the limited efficacy in the outlined studies and an increasing prevalence of aminoglycoside-resistance in *Enterobacteriaceae*, gentamicin, neomycin or tobramycin may no longer be the first choice of drugs for most promising decolonization regimens.

In our own experience different single-drug decolonization regimens were successful after a first decolonization course in 42 % of patients. Adding those patients who were successfully decolonized after a secondary/salvage regimen yielded an overall decolonization efficacy of 60 % in short-term follow-up. However, the recolonization rate of 46 % in the subgroup with available follow-up data points to a substantial proportion of patients with only temporarily negative rectal swab cultures. There are several explanations for this. Either the decolonization regimen lead to suppression of the ESBL-producing bacteria in the intestinal tract below the detection limit of the cultural method used rather than to true eradication (and renewed selection pressure due to reapplication of antimicrobial therapy in often highly comorbid patients drives ‘outgrowth’ of ESBL-E). Or the primarily successful intestinal decolonization was followed by endogenous recolonization from extraintestinal sites or exogenous recolonization from the family, hospital environment or contaminated food. Molecular methods for strain detection and typing are needed to address this important question.

When considering patients with late follow-up (>3.5 months after completion of the decolonization regimen) a sustained decolonization success was achieved in 54 %. This proportion is in the range of the above mentioned spontaneous ESBL-E clearance rate of healthy individuals, yet it may be higher than the spontaneous decolonization rate in a control group of patients with high comorbidity and repeated readmissions and still an attractive level for patients at high risk to develop symptomatic and potentially severe infection.

Whereas colistin and paromomycin have been used in diverse SDD regimens the present study is the first to investigate the impact of rifaximin in decolonization regimens. The rationale for using rifaximin were the low rates of resistance of ESBL-E along with its favourable pharmacological profile with negligible intestinal absorption yielding intraluminal or fecal rifaximin concentrations that are 50 to 500-fold higher than the MICs of *Enterobacteriaceae* [24]. Decolonization efficacy of the first line regimen tended to be higher with rifaximin compared to colistin high- or low-dose (60 % vs. 25 % or 39 %) – owing to the small number of patients included the difference proved not to be statistically significant.

The current study bears the limitations of a retrospective, non-randomized study with a small sample size not allowing for firm conclusions on decolonization efficacy. However, it is one of the very few studies that addresses specific ESBL-E decolonization strategies. In addition, by including a brief review of the existing literature/studies we wanted to highlight the methodological challenges and caveats in further studies. Another limitation is that organisms isolated from patients with failures were not systematically retested to fully assess the emergence of resistance or of non-susceptibility to decolonization and unrelated drugs. It needs to be borne in mind, however, that there are no susceptibility and resistance breakpoints for decolonization, and pre-decolonization susceptibility test results for colistin using established breakpoints, obviously, were not predictive of failure. We speculate that additional testing at high inocula and for bactericidal activity will be needed to better discriminate susceptibility versus resistance specific to intestinal decolonization. Moreover, as molecular typing of isolates of patients that failed decolonization was not performed within this study, the question of clonal diversity i.e. exogenous ESBL-E reacquisition rather than persistence under decolonization treatment remained unresolved. Finally, we used a single rectal swab followed by conventional culture in chromogenic screening medium to detect persistent intestinal ESBL-E colonization at the time of therapy initiation which may have been too insensitive or resulted in a selection bias favoring the inclusion of patients with high titers of ESBL-E. There is controversy about which method – repeated rectal swab cultures, the use of fecal samples, PCR-based detection methods – is best suited and most reliable for the detection of intestinal tract ESBL-E colonization [25–27]. Although the significance of ESBL-E carriage below the detection limit of the applied methodology is unclear, ESBL-E persistence in very low density after decolonization treatment needs to be taken into consideration.

Among the identified prerequisites for future trials we think are stringent definitions of decolonization success with long follow-up periods, reliable and sensitive sampling and detection techniques, a distinction between colonized and infected patients and between intestinal and extraintestinal colonization. Important aspects like resistance development of *Enterobacteriaceae* under prolonged decolonization treatment, achievability of sustained decolonization, identification of ESBL-E colonized patients that are at greatest risk of infection and the impact of the intestinal microbiome need to be addressed in future studies (for a detailed list see Table 3). It is only then we may be able to answer the whole range of questions that arise in the field of decolonization strategies.

## Conclusions

We conclude that the observed limited efficacy of the decolonization regimen taken together with the heterogeneity of previous studies with respect to methodology as well as interventional strategies underlines the need for randomized controlled studies in this field of eminent importance for infectious diseases and for the whole medical science.

## Abbreviations

CR-E: Carbapenem-resistent *Enterobacteriaceae*
ESBL: Extended-spectrum β-lactamases
ESBL-E: ESBL-positive *Enterobacteriaceae*
ICU: Intensive care unit
MIC: Minimal inhibitory concentration
SDD: Selective digestive tract decolonization
STEC: Shiga-toxin producing enteroaggregative *E. coli*
UTI: Urinary tract infection
